# Supplementary material for: Towards optimised extracellular vesicle proteomics from cerebrospinal fluid
Source: Sci Rep. 2023 Jun 12;13:9564. doi: 10.1038/s41598-023-36706-z (PMC10261101; doi:10.1038/s41598-023-36706-z)
Supplement: Supplementary file 1 — Supplementary Figures. [file 41598_2023_36706_MOESM1_ESM.pdf]

Supplementary figures

Towards optimised extracellular vesicle proteomics from cerebrospinal fluid

Petra Kangas, Tuula A. Nyman, Liisa Metsähonkala, Cameron Burns, Robert Tempest, Tim Williams, Jenni Karttunen* and Tarja S. Jokinen*

*Shared last author

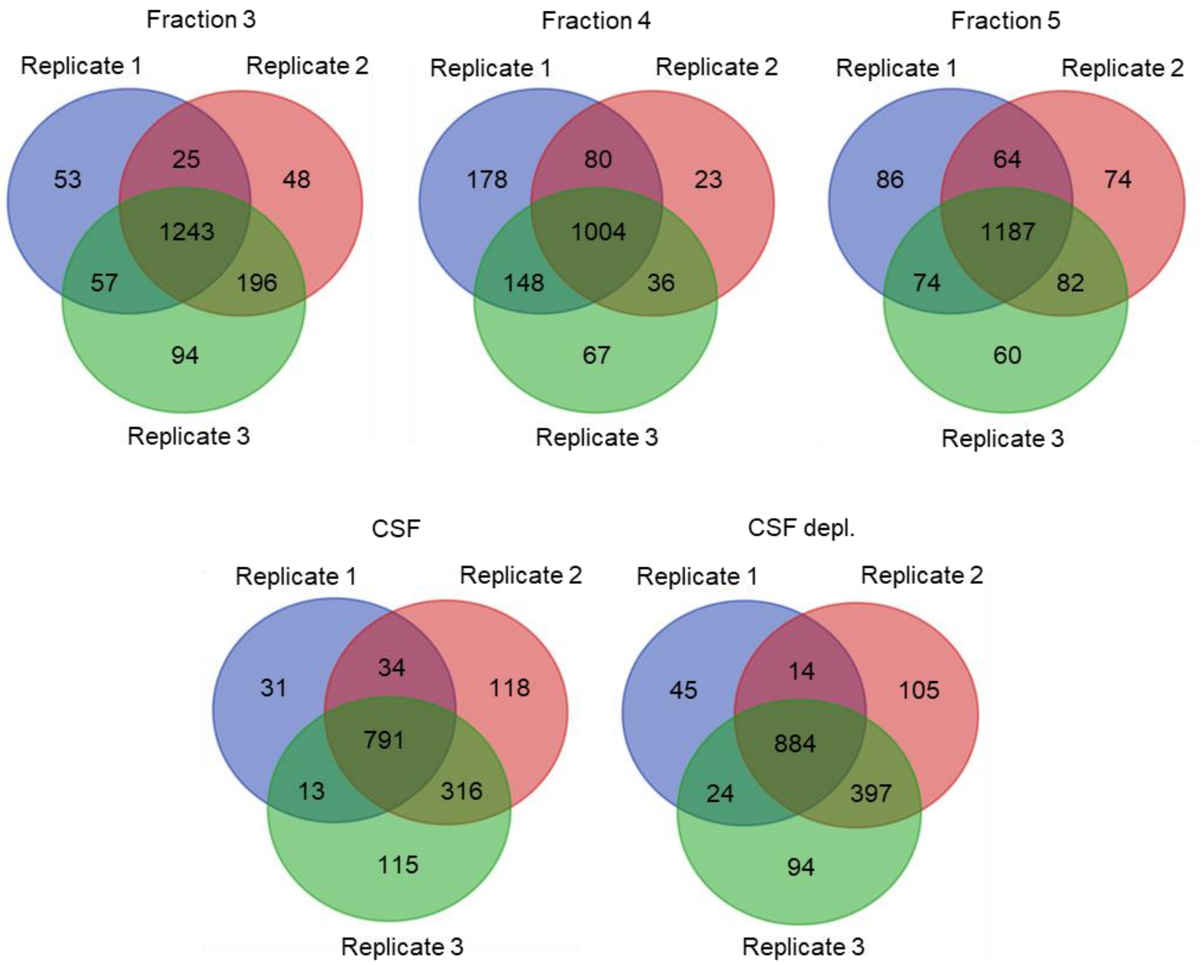

Supplementary figure S1: Venn diagrams of the identified proteins for each replicate of fractions 3-5 as well as CSF and CSF depl. Protein identification was done with 'matches between runs' active in MaxQuant. Abbreviations: CSF = cerebrospinal fluid, CSF depl. = CSF with depletion of highest abundant plasma proteins

a

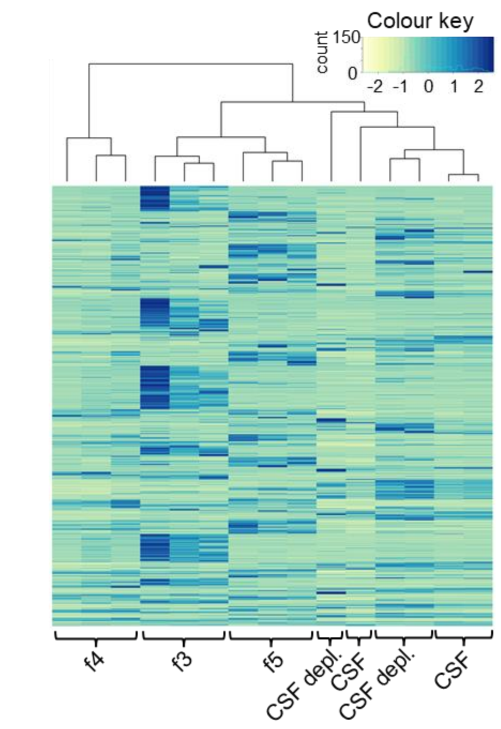

b

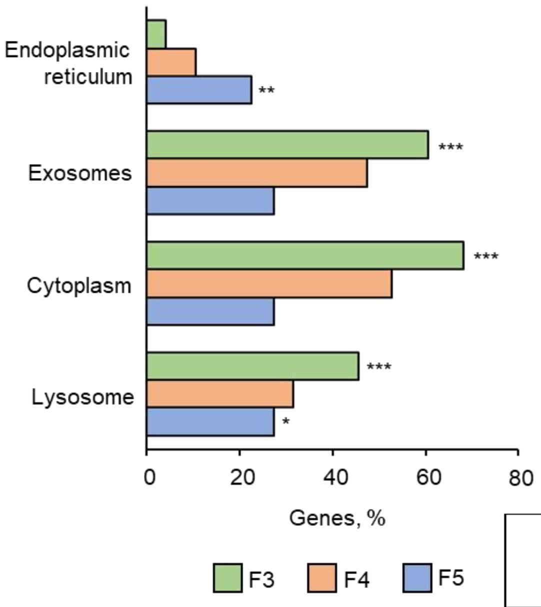

c

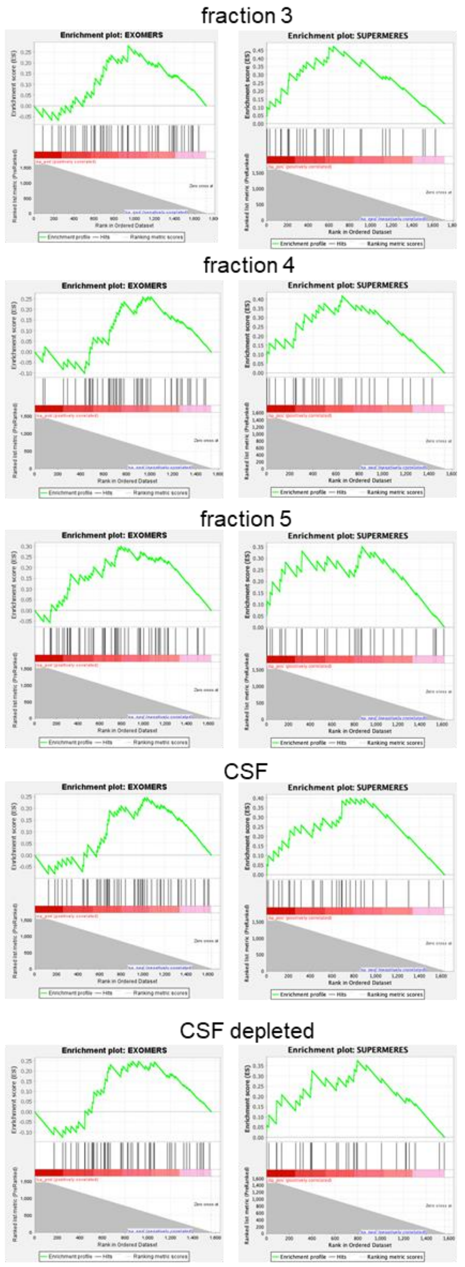

	exomers			supermeres		
	ES	NOM p-value	FDR q-val	ES	NOM p-value	FDR q-val
fraction 3	0.28	0.651	0.638	0.47	0.011	0.019
fraction 4	0.26	0.782	0.751	0.42	0.077	0.108
fraction 5	0.30	0.492	0.647	0.35	0.288	0.816
csf	0.25	0.849	0.824	0.40	0.112	0.327
csf depl.	0.24	0.844	1.0	0.38	0.231	0.612

Supplementary figure S2: a. Heatmap with clustering of all the proteins found in the samples. **b.** Pathway analysis of chosen cellular components of proteins unique to SEC fractions 3-5 using FunRich. **c.** GSEA of protein list for exomers and supermeres. Abbreviations: SEC = size-exclusion chromatography, GSEA = gene set enrichment analysis, CSF = cerebrospinal fluid, CSF depl. = CSF with depletion of highest abundant plasma proteins. *p≤0.05, **p≤0.01, ***p≤0.001.

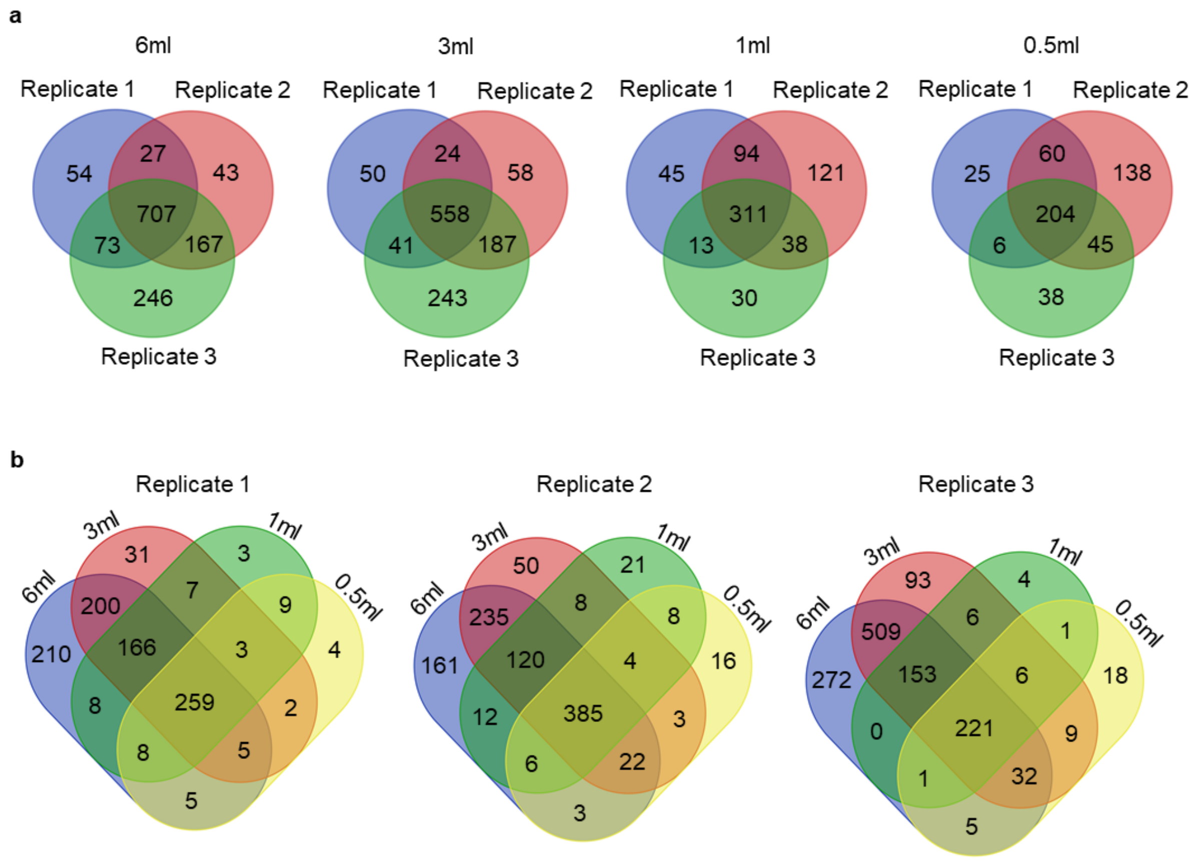

Supplementary figure S3: a. Shared proteins for the different starting volumes in each proteomics replicate. **b.** Shows the shared proteins between the different starting volumes in each proteomics replicate. Identification was done without the 'matches between runs' active in MaxQuant.

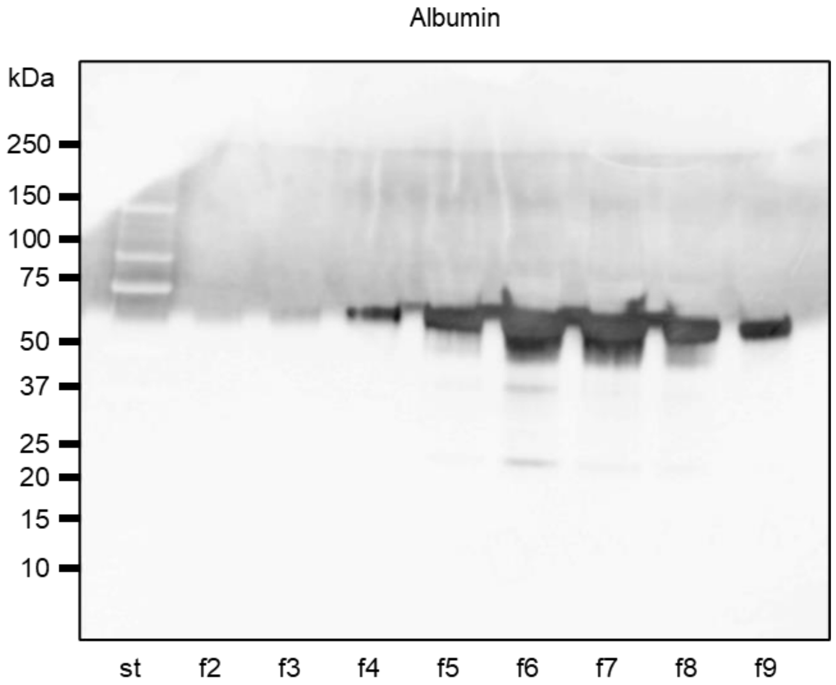

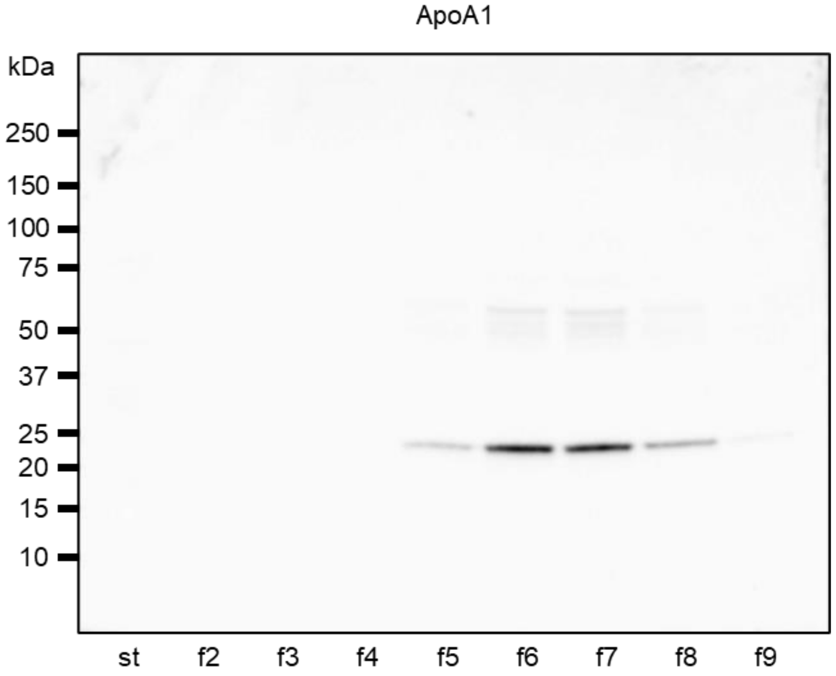

Supplementary figure S4: Uncropped Western blots of CSF SEC fractions 2-9 against Albumin and ApoA1. Abbreviations: CSF = cerebrospinal fluid, SEC = size-exclusion chromatography, st = Precision Plus Protein Dual Color Standards (#1610374, Bio-Rad Laboratories, Hercules, California, United States)

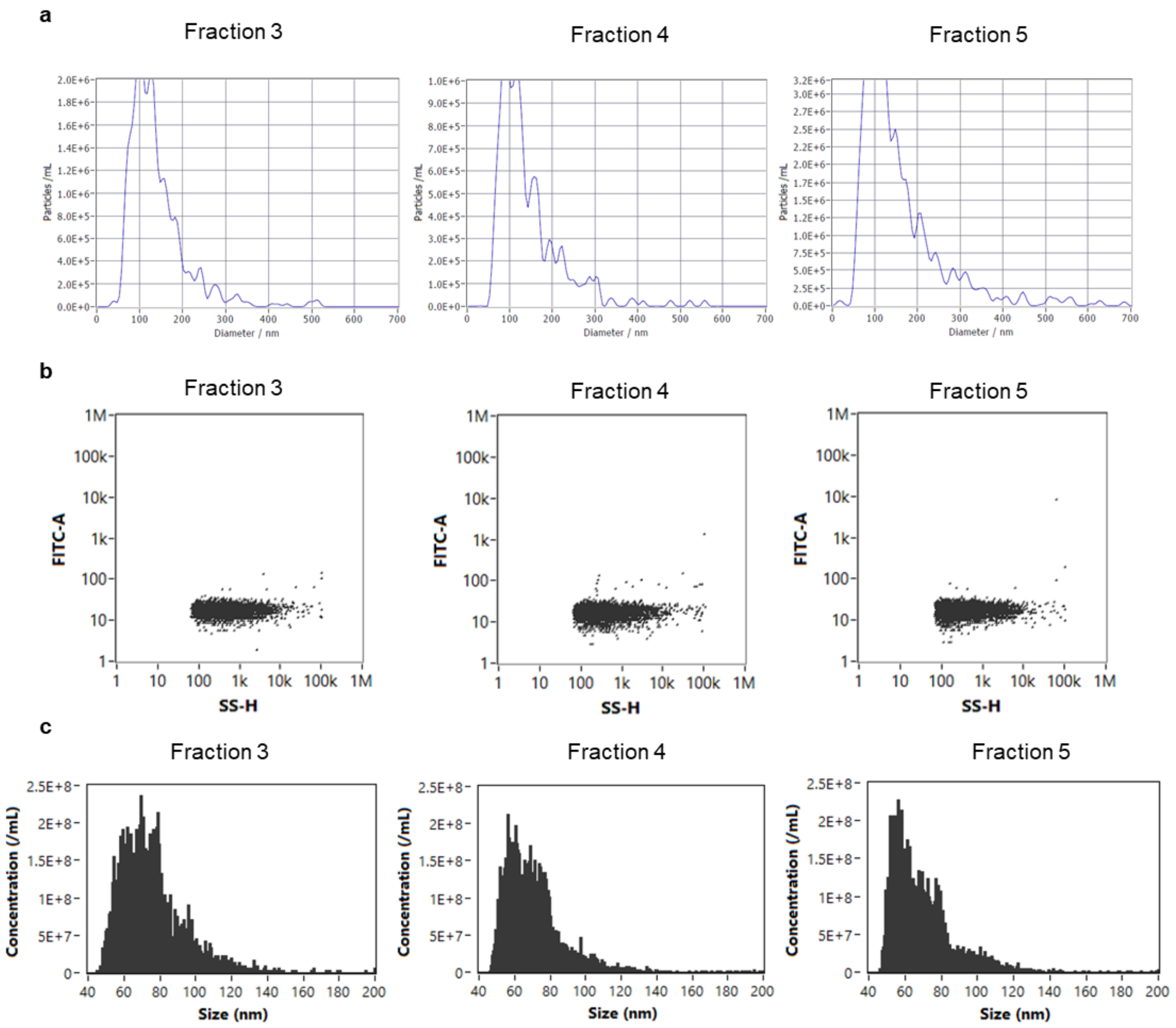

Supplementary figure S5: a. Raw data of nanoparticle analysis measurements of CSF SEC fractions 3-5. **b.** Raw data of NanoFCM measurements of CSF SEC fractions 3-5. **c.** Size and concentration raw data of CSF SEC fractions 3-5. Abbreviations: CSF = cerebrospinal fluid, SEC = size-exclusion chromatography, NanoFCM = nano-flow cytometry.
