# Supplementary material for: Towards optimised extracellular vesicle proteomics from cerebrospinal fluid
Source: Sci Rep. 2023 Jun 12;13:9564. doi: 10.1038/s41598-023-36706-z (PMC10261101; doi:10.1038/s41598-023-36706-z)
Supplement: Supplementary file 3 — Supplementary Information. [file 41598_2023_36706_MOESM3_ESM.pdf]

Supplementary material

Towards optimised extracellular vesicle proteomics from cerebrospinal fluid

Petra Kangas, Tuula A. Nyman, Liisa Metsähonkala, Cameron Burns, Robert Tempest, Tim Williams, Jenni Karttunen* and Tarja S. Jokinen*

*Shared last author

Supplementary file S1: Articles analysed for the literature review.

Akers, J. C. *et al.* A cerebrospinal fluid microRNA signature as biomarker for glioblastoma. *Oncotarget* **8**, 68769–68779 (2017).

Akers, J. C. *et al.* miRNA contents of cerebrospinal fluid extracellular vesicles in glioblastoma patients. *J Neurooncol* **123**, 205–216 (2015).

Akers, J. C. *et al.* Comparative Analysis of Technologies for Quantifying Extracellular Vesicles (EVs) in Clinical Cerebrospinal Fluids (CSF). *PLoS ONE* **11**, e0149866 (2016).

Akers, J. C. *et al.* Optimizing preservation of extracellular vesicular miRNAs derived from clinical cerebrospinal fluid. *CBM* **17**, 125–132 (2016).

Alsop, E. *et al.* A Novel Tissue Atlas and Online Tool for the Interrogation of Small RNA Expression in Human Tissues and Biofluids. *Front. Cell Dev. Biol.* **10**, 804164 (2022).

Anderson, M. R. *et al.* Viral antigens detectable in CSF exosomes from patients with retrovirus associated neurologic disease: functional role of exosomes. *Clinical and Translational Medicine* **7**, (2018).

Balusu, S. *et al.* Identification of a novel mechanism of blood–brain communication during peripheral inflammation via choroid plexus-derived extracellular vesicles. *EMBO Mol Med* **8**, 1162–1183 (2016).

Castañeyra-Ruiz, L. *et al.* AQP4 labels a subpopulation of white matter-dependent glial radial cells affected by pediatric hydrocephalus, and its expression increased in glial microvesicles released to the cerebrospinal fluid in obstructive hydrocephalus. *acta neuropathol commun* **10**, 41 (2022).

Cheng, P. *et al.* Detection and significance of exosomal mRNA expression profiles in the cerebrospinal fluid of patients with meningeal carcinomatosis. *J Mol Neurosci* **71**, 790–803 (2021).

Colombo, F. *et al.* Cytokines Stimulate the Release of Microvesicles from Myeloid Cells Independently from the P2X7 Receptor/Acid Sphingomyelinase Pathway. *Front. Immunol.* **9**, 204 (2018).

Costa, J. *et al.* Investigating LGALS3BP/90 K glycoprotein in the cerebrospinal fluid of patients with neurological diseases. *Sci Rep* **10**, 5649 (2020).

Cressatti, M. *et al.* Characterization and heme oxygenase-1 content of extracellular vesicles in human biofluids. *J. Neurochem.* **157**, 2195–2209 (2021).

Crotti, A. *et al.* BIN1 favors the spreading of Tau via extracellular vesicles. *Sci Rep* **9**, 9477 (2019).
da Cruz, A. B. *et al.* Human extracellular vesicles and correlation with two clinical forms of toxoplasmosis. *PLoS ONE* **15**, e0229602 (2020).
Daaboul, G. G. *et al.* Digital Detection of Exosomes by Interferometric Imaging. *Sci Rep* **6**, 37246 (2016).
Dalla Costa, G. *et al.* CSF extracellular vesicles and risk of disease activity after a first demyelinating event. *Mult Scler* **27**, 1606–1610 (2021).
de Rivero Vaccari, J. P. *et al.* Exosome-mediated inflammasome signaling after central nervous system injury. *J. Neurochem.* **136**, 39–48 (2016).
Derkow, K. *et al.* Distinct expression of the neurotoxic microRNA family let-7 in the cerebrospinal fluid of patients with Alzheimer's disease. *PLoS ONE* **13**, e0200602 (2018).
Ding, X. *et al.* Exposure to ALS-FTD-CSF generates TDP-43 aggregates in glioblastoma cells through exosomes and TNTs-like structure. *Oncotarget* **6**, 24178–24191 (2015).
Dozio, V. *et al.* Cerebrospinal Fluid-Derived Microvesicles From Sleeping Sickness Patients Alter Protein Expression in Human Astrocytes. *Front. Cell. Infect. Microbiol.* **9**, 391 (2019).
Egyed, B. *et al.* MicroRNA-181a as novel liquid biopsy marker of central nervous system involvement in pediatric acute lymphoblastic leukemia. *J Transl Med* **18**, 250 (2020).
Eitan, E. *et al.* Extracellular vesicle-associated Aβ mediates trans-neuronal bioenergetic and Ca2+-handling deficits in Alzheimer's disease models. *npj Aging Mech Dis* **2**, 16019 (2016).
Elkouris, M. *et al.* Long Non-coding RNAs Associated With Neurodegeneration-Linked Genes Are Reduced in Parkinson's Disease Patients. *Front. Cell. Neurosci.* **13**, 58 (2019).
Emelyanov, A. *et al.* Cryo-electron microscopy of extracellular vesicles from cerebrospinal fluid. *PLoS ONE* **15**, e0227949 (2020).
Figueroa, J. M. *et al.* Detection of wild-type EGFR amplification and EGFRvIII mutation in CSF-derived extracellular vesicles of glioblastoma patients. *Neuro-Oncology* **19**, 1494–1502 (2017).
Galazka, G., Mycko, M. P., Selmaj, I., Raine, C. S. & Selmaj, K. W. Multiple sclerosis: Serum-derived exosomes express myelin proteins. *Mult Scler* **24**, 449–458 (2018).
Gelibter, S. *et al.* Spinal Fluid Myeloid Microvesicles Predict Disease Course in Multiple Sclerosis. *Ann Neurol* **90**, 253–265 (2021).
Geraci, F. *et al.* Differences in Intercellular Communication During Clinical Relapse and Gadolinium-Enhanced MRI in Patients With Relapsing Remitting Multiple Sclerosis: A Study of the Composition of Extracellular Vesicles in Cerebrospinal Fluid. *Front. Cell. Neurosci.* **12**, 418 (2018).
Gomes de Andrade, G. *et al.* The Aging Process Alters IL-1β and CD63 Levels Differently in Extracellular Vesicles Obtained from the Plasma and Cerebrospinal Fluid. *Neuroimmunomodulation* **25**, 18–22 (2018).
Goswami, S. *et al.* Differential Expression and Significance of Circulating microRNAs in Cerebrospinal Fluid of Acute Encephalitis Patients Infected with Japanese Encephalitis Virus. *Mol Neurobiol* **54**, 1541–1551 (2017).
Gu, J. *et al.* Exosomes expressing neuronal autoantigens induced immune response in antibody-positive autoimmune encephalitis. *Molecular Immunology* **131**, 164–170 (2021).
Guha, D. *et al.* Proteomic analysis of cerebrospinal fluid extracellular vesicles reveals synaptic injury, inflammation, and stress response markers in HIV patients with cognitive impairment. *J Neuroinflammation* **16**, 254 (2019).

Guha, D. *et al.* Cerebrospinal fluid extracellular vesicles and neurofilament light protein as biomarkers of central nervous system injury in HIV-infected patients on antiretroviral therapy. *AIDS* **33**, 615–625 (2019).
Gui, Y., Liu, H., Zhang, L., Lv, W. & Hu, X. Altered microRNA profiles in cerebrospinal fluid exosome in Parkinson disease and Alzheimer disease. *Oncotarget* **6**, 37043–37053 (2015).
Guix, F. *et al.* Detection of Aggregation-Competent Tau in Neuron-Derived Extracellular Vesicles. *IJMS* **19**, 663 (2018).
Hayashi, N. *et al.* Proteomic analysis of exosome-enriched fractions derived from cerebrospinal fluid of amyotrophic lateral sclerosis patients. *Neuroscience Research* **160**, 43–49 (2020).
He, J. *et al.* Exosomal Circular RNA as a Biomarker Platform for the Early Diagnosis of Immune-Mediated Demyelinating Disease. *Front. Genet.* **10**, 860 (2019).
Henderson, L. J. *et al.* Presence of Tat and transactivation response element in spinal fluid despite antiretroviral therapy. *AIDS* **33**, S145–S157 (2019).
Hirschberg, Y. *et al.* Characterising extracellular vesicles from individual low volume cerebrospinal fluid samples, isolated by SmartSEC. *J of Extracellular Bio* **1**, (2022).
Hong, Z. *et al.* Development of a Sensitive Diagnostic Assay for Parkinson Disease Quantifying α-Synuclein–Containing Extracellular Vesicles. *Neurology* **96**, e2332–e2345 (2021).
Hou, X. *et al.* Identification of a potential exosomal biomarker in spinocerebellar ataxia Type 3/Machado–Joseph disease. *Epigenomics* **11**, 1037–1056 (2019).
Huang, M. *et al.* Bioinformatic Analysis of Exosomal MicroRNAs of Cerebrospinal Fluid in Ischemic Stroke Rats After Physical Exercise. *Neurochem Res* **46**, 1540–1553 (2021).
Jain, G. *et al.* A combined miRNA–piRNA signature to detect Alzheimer’s disease. *Transl Psychiatry* **9**, 250 (2019).
Kim, S. H., Yun, S.-W., Kim, H. R. & Chae, S. A. Exosomal microRNA expression profiles of cerebrospinal fluid in febrile seizure patients. *Seizure* **81**, 47–52 (2020).
Kong, F.-L., Wang, X.-P., Li, Y.-N. & Wang, H.-X. The role of exosomes derived from cerebrospinal fluid of spinal cord injury in neuron proliferation *in vitro*. *Artificial Cells, Nanomedicine, and Biotechnology* **46**, 200–205 (2018).
Krušić Alić, V. *et al.* Extracellular Vesicles from Human Cerebrospinal Fluid Are Effectively Separated by Sepharose CL-6B—Comparison of Four Gravity-Flow Size Exclusion Chromatography Methods. *Biomedicines* **10**, 785 (2022).
Kuharić, J. *et al.* Severe Traumatic Brain Injury Induces Early Changes in the Physical Properties and Protein Composition of Intracranial Extracellular Vesicles. *Journal of Neurotrauma* **36**, 190–200 (2019).
Kurzawa-Akanbi, M. *et al.* Altered ceramide metabolism is a feature in the extracellular vesicle-mediated spread of alpha-synuclein in Lewy body disorders. *Acta Neuropathol* **142**, 961–984 (2021).
Lee, J. *et al.* Exosomal proteome analysis of cerebrospinal fluid detects biosignatures of neuromyelitis optica and multiple sclerosis. *Clinica Chimica Acta* **462**, 118–126 (2016).
Lee, K.-Y. *et al.* Nanoparticles in 472 Human Cerebrospinal Fluid: Changes in Extracellular Vesicle Concentration and miR-21 Expression as a Biomarker for Leptomeningeal Metastasis. *Cancers* **12**, 2745 (2020).
Lee, K.-Y. *et al.* Molecular Signature of Extracellular Vesicular Small Non-Coding RNAs Derived from Cerebrospinal Fluid of Leptomeningeal Metastasis Patients: Functional Implication of miR-21 and Other Small RNAs in Cancer Malignancy. *Cancers* **13**, 209 (2021).

Lepko, T. *et al.* Choroid plexus-derived miR-204 regulates the number of quiescent neural stem cells in the adult brain. *EMBO J* **38**, (2019).

Li, D.-B. *et al.* Plasma Exosomal miRNA-122-5p and miR-300-3p as Potential Markers for Transient Ischaemic Attack in Rats. *Front. Aging Neurosci.* **10**, 24 (2018).

Li, J. *et al.* Microvesicles shed from microglia activated by the P2X7-p38 pathway are involved in neuropathic pain induced by spinal nerve ligation in rats. *Purinergic Signalling* **13**, 13–26 (2017).

Li, J., Yuan, H., Xu, H., Zhao, H. & Xiong, N. Hypoxic Cancer-Secreted Exosomal miR-182-5p Promotes Glioblastoma Angiogenesis by Targeting Kruppel-like Factor 2 and 4. *Molecular Cancer Research* **18**, 1218–1231 (2020).

Li, M. *et al.* Isolation of Exosome Nanoparticles from Human Cerebrospinal Fluid for Proteomic Analysis. *ACS Appl. Nano Mater.* **4**, 3351–3359 (2021).

Li, Y. *et al.* Cerebrospinal Fluid Extracellular Vesicles with Distinct Properties in Autoimmune Encephalitis and Herpes Simplex Encephalitis. *Mol Neurobiol* **59**, 2441–2455 (2022).

Li, Y. *et al.* EV-origin: Enumerating the tissue-cellular origin of circulating extracellular vesicles using exLR profile. *Computational and Structural Biotechnology Journal* **18**, 2851–2859 (2020).

Lin, Y.-W. *et al.* Regulation of exosome secretion by cellular retinoic acid binding protein 1 contributes to systemic anti-inflammation. *Cell Commun Signal* **19**, 69 (2021).

Liu, C. G. *et al.* MicroRNA-135a in ABCA1-labeled Exosome is a Serum Biomarker Candidate for Alzheimer's Disease. *Biomedical and Environmental Sciences* **34**, 19–28 (2021).

Liu, C.-G., Zhao, Y., Lu, Y. & Wang, P.-C. ABCA1-Labeled Exosomes in Serum Contain Higher MicroRNA-193b Levels in Alzheimer's Disease. *BioMed Research International* **2021**, 1–10 (2021).

Liu, X. *et al.* Inhibition of P2X7 receptors improves outcomes after traumatic brain injury in rats. *Purinergic Signalling* **13**, 529–544 (2017).

Longobardi, A. *et al.* Cerebrospinal Fluid EV Concentration and Size Are Altered in Alzheimer's Disease and Dementia with Lewy Bodies. *Cells* **11**, 462 (2022).

López-Pérez, Ó. *et al.* Cerebrospinal Fluid and Plasma Small Extracellular Vesicles and miRNAs as Biomarkers for Prion Diseases. *IJMS* **22**, 6822 (2021).

Luo, X. *et al.* Plasma Exosomal miR-450b-5p as a Possible Biomarker and Therapeutic Target for Transient Ischaemic Attacks in Rats. *J Mol Neurosci* **69**, 516–526 (2019).

Madhankumar, A. B. *et al.* Interleukin-13 conjugated quantum dots for identification of glioma initiating cells and their extracellular vesicles. *Acta Biomaterialia* **58**, 205–213 (2017).

Manek, R. *et al.* Protein Biomarkers and Neuroproteomics Characterization of Microvesicles/Exosomes from Human Cerebrospinal Fluid Following Traumatic Brain Injury. *Mol Neurobiol* **55**, 6112–6128 (2018).

Masvekar, R., Mizrahi, J., Park, J., Williamson, P. R. & Bielekova, B. Quantifications of CSF Apoptotic Bodies Do Not Provide Clinical Value in Multiple Sclerosis. *Front. Neurol.* **10**, 1241 (2019).

McKeever, P. M. *et al.* MicroRNA Expression Levels Are Altered in the Cerebrospinal Fluid of Patients with Young-Onset Alzheimer's Disease. *Mol Neurobiol* **55**, 8826–8841 (2018).

Minakaki, G. *et al.* Autophagy inhibition promotes SNCA/alpha-synuclein release and transfer via extracellular vesicles with a hybrid autophagosome-exosome-like phenotype. *Autophagy* **14**, 98–119 (2018).

Muraoka, S. *et al.* Proteomic Profiling of Extracellular Vesicles Isolated From Cerebrospinal Fluid of Former National Football League Players at Risk for Chronic Traumatic Encephalopathy. *Front. Neurosci.* **13**, 1059 (2019).

Muraoka, S. *et al.* Proteomic Profiling of Extracellular Vesicles Derived from Cerebrospinal Fluid of Alzheimer's Disease Patients: A Pilot Study. *Cells* **9**, 1959 (2020).

Norman, M. *et al.* L1CAM is not associated with extracellular vesicles in human cerebrospinal fluid or plasma. *Nat Methods* **18**, 631–634 (2021).

Otake, K., Kamiguchi, H. & Hirozane, Y. Identification of biomarkers for amyotrophic lateral sclerosis by comprehensive analysis of exosomal mRNAs in human cerebrospinal fluid. *BMC Med Genomics* **12**, 7 (2019).

Pieragostino, D. *et al.* Enhanced release of acid sphingomyelinase-enriched exosomes generates a lipidomics signature in CSF of Multiple Sclerosis patients. *Sci Rep* **8**, 3071 (2018).

Pieragostino, D. *et al.* Proteomics characterization of extracellular vesicles sorted by flow cytometry reveals a disease-specific molecular cross-talk from cerebrospinal fluid and tears in multiple sclerosis. *Journal of Proteomics* **204**, 103403 (2019).

Pisa, M. *et al.* Subclinical anterior optic pathway involvement in early multiple sclerosis and clinically isolated syndromes. *Brain* **144**, 848–862 (2021).

Prada, I. *et al.* Glia-to-neuron transfer of miRNAs via extracellular vesicles: a new mechanism underlying inflammation-induced synaptic alterations. *Acta Neuropathol* **135**, 529–550 (2018).

Prieto-Fernández, E. *et al.* A Comprehensive Study of Vesicular and Non-Vesicular miRNAs from a Volume of Cerebrospinal Fluid Compatible with Clinical Practice. *Theranostics* **9**, 4567–4579 (2019).

Qi, Y. *et al.* The dual role of glioma exosomal microRNAs: glioma eliminates tumor suppressor miR-1298-5p via exosomes to promote immunosuppressive effects of MDSCs. *Cell Death Dis* **13**, 426 (2022).

Qiu, W. *et al.* Exosomal miR-1246 from glioma patient body fluids drives the differentiation and activation of myeloid-derived suppressor cells. *Molecular Therapy* **29**, 3449–3464 (2021).

Ragonese, P. *et al.* Toxic effects on astrocytes of extracellular vesicles from CSF of multiple sclerosis patients: a pilot in vitro study. *pjp* **71**, 270–276 (2020).

Raoof, R. *et al.* Cerebrospinal fluid microRNAs are potential biomarkers of temporal lobe epilepsy and status epilepticus. *Sci Rep* **7**, 3328 (2017).

Rather, H. A. *et al.* Mass Spectrometry-Based Proteome Profiling of Extracellular Vesicles Derived from the Cerebrospinal Fluid of Adult Rhesus Monkeys Exposed to Cocaine throughout Gestation. *Biomolecules* **12**, 510 (2022).

Raval, A. P., Martinez, C. C., Mejias, N. H. & de Rivero Vaccari, J. P. Sexual dimorphism in inflammasome-containing extracellular vesicles and the regulation of innate immunity in the brain of reproductive senescent females. *Neurochemistry International* **127**, 29–37 (2019).

Riancho, J. *et al.* MicroRNA Profile in Patients with Alzheimer's Disease: Analysis of miR-9-5p and miR-598 in Raw and Exosome Enriched Cerebrospinal Fluid Samples. *JAD* **57**, 483–491 (2017).

Rider, M. A., Hurwitz, S. N. & Meckes, D. G. ExtraPEG: A Polyethylene Glycol-Based Method for Enrichment of Extracellular Vesicles. *Sci Rep* **6**, 23978 (2016).

Sanchez, I. I. *et al.* Huntington's disease mice and human brain tissue exhibit increased G3BP1 granules and TDP43 mislocalization. *Journal of Clinical Investigation* **131**, e140723 (2021).

Sandau, U. S. *et al.* Differential Effects of APOE Genotype on MicroRNA Cargo of Cerebrospinal Fluid Extracellular Vesicles in Females With Alzheimer's Disease Compared to Males. *Front. Cell Dev. Biol.* **10**, 864022 (2022).

Saugstad, J. A. *et al.* Analysis of extracellular RNA in cerebrospinal fluid. *Journal of Extracellular Vesicles* **6**, 1317577 (2017).

Schneider, R. *et al.* Downregulation of exosomal miR-204-5p and miR-632 as a biomarker for FTD: a GENFI study. *J Neurol Neurosurg Psychiatry* **89**, 851–858 (2018).

Shi, R. *et al.* Exosomal levels of miRNA-21 from cerebrospinal fluids associated with poor prognosis and tumor recurrence of glioma patients. *Oncotarget* **6**, 26971–26981 (2015).

Sjoqvist, S. & Otake, K. A pilot study using proximity extension assay of cerebrospinal fluid and its extracellular vesicles identifies novel amyotrophic lateral sclerosis biomarker candidates. *Biochemical and Biophysical Research Communications* **613**, 166–173 (2022).

Sjoqvist, S., Otake, K. & Hirozane, Y. Analysis of Cerebrospinal Fluid Extracellular Vesicles by Proximity Extension Assay: A Comparative Study of Four Isolation Kits. *IJMS* **21**, 9425 (2020).

Skalnikova *et al.* Isolation and Characterization of Small Extracellular Vesicles from Porcine Blood Plasma, Cerebrospinal Fluid, and Seminal Plasma. *Proteomes* **7**, 17 (2019).

Soares Martins, T., Catita, J., Martins Rosa, I., A. B. da Cruz e Silva, O. & Henriques, A. G. Exosome isolation from distinct biofluids using precipitation and column-based approaches. *PLoS ONE* **13**, e0198820 (2018).

Spaull, R. *et al.* Exosomes populate the cerebrospinal fluid of preterm infants with post-haemorrhagic hydrocephalus. *Int. j. dev. neurosci.* **73**, 59–65 (2019).

Spitzer, P. *et al.* Microvesicles from cerebrospinal fluid of patients with Alzheimer's disease display reduced concentrations of tau and APP protein. *Sci Rep* **9**, 7089 (2019).

Stuendl, A. *et al.* Induction of α-synuclein aggregate formation by CSF exosomes from patients with Parkinson's disease and dementia with Lewy bodies. *Brain* **139**, 481–494 (2016).

Tan, N., Hu, S., Hu, Z., Wu, Z. & Wang, B. Quantitative proteomic characterization of microvesicles/exosomes from the cerebrospinal fluid of patients with acute bilirubin encephalopathy. *Mol Med Rep* **22**, 1257–1268 (2020).

Tan, Y. J. *et al.* Altered Cerebrospinal Fluid Exosomal microRNA Levels in Young-Onset Alzheimer's Disease and Frontotemporal Dementia. *ADR* **5**, 805–813 (2021).

Ter-Ovanesyan, D. *et al.* Framework for rapid comparison of extracellular vesicle isolation methods. *eLife* **10**, e70725 (2021).

Thakur, A. *et al.* In vivo liquid biopsy for glioblastoma malignancy by the AFM and LSPR based sensing of exosomal CD44 and CD133 in a mouse model. *Biosensors and Bioelectronics* **191**, 113476 (2021).

Thompson, A. G. *et al.* UFLC-Derived CSF Extracellular Vesicle Origin and Proteome. *Proteomics* 1800257 (2018) doi:10.1002/pmic.201800257.

Thompson, A. G. *et al.* CSF extracellular vesicle proteomics demonstrates altered protein homeostasis in amyotrophic lateral sclerosis. *Clin Proteom* **17**, 31 (2020).

Tsutsui, T. *et al.* Glioma-derived extracellular vesicles promote tumor progression by conveying WT1. *Carcinogenesis* **41**, 1238–1245 (2020).

Utz, J. *et al.* Cerebrospinal Fluid of Patients With Alzheimer's Disease Contains Increased Percentages of Synaptophysin-Bearing Microvesicles. *Front. Aging Neurosci.* **13**, 682115 (2021).

Vacchi, E. *et al.* Profiling Inflammatory Extracellular Vesicles in Plasma and Cerebrospinal Fluid: An Optimized Diagnostic Model for Parkinson's Disease. *Biomedicines* **9**, 230 (2021).

Van Hoecke, L. *et al.* Involvement of the Choroid Plexus in the Pathogenesis of Niemann-Pick Disease Type C. *Front. Cell. Neurosci.* **15**, 757482 (2021).

Vandendriessche, C. *et al.* Importance of extracellular vesicle secretion at the blood–cerebrospinal fluid interface in the pathogenesis of Alzheimer's disease. *acta neuropathol commun* **9**, 143 (2021).

Wang, G. *et al.* A Panel of Exosome-Derived miRNAs of Cerebrospinal Fluid for the Diagnosis of Moyamoya Disease. *Front. Neurosci.* **14**, 548278 (2020).

Wang, M. *et al.* Exosomal LGALS9 in the cerebrospinal fluid of glioblastoma patients suppressed dendritic cell antigen presentation and cytotoxic T-cell immunity. *Cell Death Dis* **11**, 896 (2020).

Wang, S., Kelly, K., Brotchie, J. M., Koprich, J. B. & West, A. B. Exosome markers of LRRK2 kinase inhibition. *npj Parkinsons Dis.* **6**, 32 (2020).

Wang, S. *et al.* Elevated LRRK2 autophosphorylation in brain-derived and peripheral exosomes in LRRK2 mutation carriers. *acta neuropathol commun* **5**, 86 (2017).

Wang, Y. *et al.* The release and trans-synaptic transmission of Tau via exosomes. *Mol Neurodegeneration* **12**, 5 (2017).

Wei, Z. *et al.* Coding and noncoding landscape of extracellular RNA released by human glioma stem cells. *Nat Commun* **8**, 1145 (2017).

Welton, J. L. *et al.* Cerebrospinal fluid extracellular vesicle enrichment for protein biomarker discovery in neurological disease; multiple sclerosis. *Journal of Extracellular Vesicles* **6**, 1369805 (2017).

Wilson, M. E. *et al.* Coagulation parameters following equine herpesvirus type 1 infection in horses. *Equine Vet J* **51**, 102–107 (2019).

Xu, H. *et al.* miR-3184-3p enriched in cerebrospinal fluid exosomes contributes to progression of glioma and promotes M2-like macrophage polarization. *Cancer Science* cas.15372 (2022) doi:10.1111/cas.15372.

Yagi, Y. *et al.* Next-generation sequencing-based small RNA profiling of cerebrospinal fluid exosomes. *Neuroscience Letters* **636**, 48–57 (2017).

Yang, Y. *et al.* Cerebrospinal Fluid Particles in Alzheimer Disease and Parkinson Disease. *J Neuropathol Exp Neurol* **74**, 672–687 (2015).

Yao, Y. *et al.* Interleukin-6 in Cerebrospinal Fluid Small Extracellular Vesicles as a Potential Biomarker for Prognosis of Aneurysmal Subarachnoid Haemorrhage. *NDT* **Volume 17**, 1423–1431 (2021).

Yelick, J. *et al.* Elevated exosomal secretion of miR-124-3p from spinal neurons positively associates with disease severity in ALS. *Experimental Neurology* **333**, 113414 (2020).
